# Neural Basis of Anticipatory Multisensory Integration

**DOI:** 10.3390/brainsci11070843

**Published:** 2021-06-25

**Authors:** Linda Fiorini, Marika Berchicci, Elena Mussini, Valentina Bianco, Stefania Lucia, Francesco Di Russo

**Affiliations:** 1 Department of Movement, Human and Health Sciences, University of Rome “Foro Italico”, 00135 Rome, Italy; m.berchicci@gmail.com (M.B.); elena.mussini@outlook.com (E.M.); biancovalentina86@gmail.com (V.B.); stefi.lucia7@gmail.com (S.L.); francesco.dirusso@uniroma4.it (F.D.R.); 2 Department of Psychology, University of Rome “La Sapienza”, 00185 Rome, Italy; 3 University “G. d’Annunzio” of Chieti-Pescara, 66100 Chieti, Italy; 4 Department of Languages and Literatures, Communication, Education and Society, University of Udine, 33100 Udine, Italy; 5 IRCCS Fondazione Santa Lucia, 00179 Rome, Italy

**Keywords:** multisensory perception, passive perception, ERP, visual, auditory, sensory preparation

## Abstract

The brain is able to gather different sensory information to enhance salient event perception, thus yielding a unified perceptual experience of multisensory events. Multisensory integration has been widely studied, and the literature supports the hypothesis that it can occur across various stages of stimulus processing, including both bottom-up and top-down control. However, evidence on anticipatory multisensory integration occurring in the fore period preceding the presentation of the expected stimulus in passive tasks, is missing. By means of event-related potentials (ERPs), it has been recently proposed that visual and auditory unimodal stimulations are preceded by sensory-specific readiness activities. Accordingly, in the present study, we tested the occurrence of multisensory integration in the endogenous anticipatory phase of sensory processing, combining visual and auditory stimuli during unimodal and multimodal passive ERP paradigms. Results showed that the modality-specific pre-stimulus ERP components (i.e., the auditory positivity -aP- and the visual negativity -vN-) started earlier and were larger in the multimodal stimulation compared with the sum of the ERPs elicited by the unimodal stimulations. The same amplitude effect was also present for the early auditory N1 and visual P1 components. This anticipatory multisensory effect seems to influence stimulus processing, boosting the magnitude of early stimulus processing. This paves the way for new perspectives on the neural basis of multisensory integration.

## 1. Introduction

The human brain continuously integrates information coming from different sensory modalities to create a coherent and unified perception of the world. This multisensory integration usually boosts perception, as in the case of language understanding, which is improved when auditory information is integrated with visual cues, such as facial expressions or other body movements [1].

Although for many years it was widely accepted that multisensory integration (or binding) would take place in higher cortical areas specialized for this purpose i.e., the superior temporal sulcus [2,3,4] and the intraparietal sulcus [5], and at relatively late stages of sensory codification, recent studies instead suggest that multisensory processing occurs at early stages of sensory processing, also in modality-specific cortices. This upstream idea on multisensory integration was initially proposed by Stein and his colleagues [6], who found bimodal neurons in the superior colliculus of cats [7]. Later, several electrophysiological studies on human brains showed that multisensory interaction takes place even in unimodal areas of sensory cortices in early processing phases, as indexed by the auditory N1 component and the visual P1 component, peaking approximately 100 ms after stimulus onset [8,9,10,11,12,13,14,15,16]. Neuroimaging studies confirmed the activation of primary sensory cortices when stimuli from a different sensory modality were presented [17,18,19]. Collectively, these findings demonstrate that multimodal stimulation facilitates task performance because of more intense activity in sensory cortices than unimodal stimulation alone. The multimodal effect on auditory (A) and visual (V) stimulus processing can be expressed by the equation AV > A + V, where AV indicates the simultaneous presentation of A and V stimuli. This equation suggests that multisensory processing is likely governed by both bottom-up, sensory–sensory interactions and top-down brain mechanisms, whose interactions allow for the selection, amplification and integration of sensory input [14]. However, while it is widely accepted that these multimodal functions can modulate the neural responses evoked and followed by sensory stimuli, it is still unknown if these multisensory mechanisms may also act at the level of sensory preparation before stimuli appearance. This sensory readiness may be intended as a pre-activation of the primary sensory cortex in preparation for an expected stimulus. Indeed, in line with both the thalamic gating theory [20,21] and the threshold regulation theory [22,23], endogenous sensory-specific anticipatory activities are processed in modality-specific cortical areas [24].

In recent studies, the present research group observed the electrophysiological correlates of sensory preparation; in particular, modality-specific event-related potentials (ERPs) started almost one second before stimulus onset, even during passive sensorial stimulation [25,26]. These slow ERP components were labelled visual negativity (vN) for the visual modality, auditory positivity (aP) for the auditory modality, and somatosensory negativity (sN) for the somatosensory modality. The vN is a negative wave with a focus of activity over bilateral parieto-occipital scalp areas that has been localized in extrastriate cortices. The aP is a positive wave with central-medial distribution that has been localized in the auditory cortex. The sN is a negative wave with parietal distribution, contralateral to the stimulated hand, that has been localized in the somatosensory cortex. These components seem to reflect pre-stimulus top-down functions, facilitating the following bottom-up post-stimulus processing, as reflected by the correlation between the vN amplitude and the response time in visuomotor tasks [26]. Gender differences on the vN were also proposed [25,27,28].

Considering the abovementioned evidence of anticipatory ERPs associated with unimodal stimulations, it appears crucial to strive for a deeper understanding of multisensory interaction investigating the anticipatory phase. Therefore, the aim of the present study is to unveil audio-visual multisensory interactions for upcoming events, analysing the vN and the aP components. To accomplish this aim, we implemented a passive paradigm design, with the unique instruction for participants to look at a fixation point during stimuli presentation. We collected three conditions: the unisensory auditory (A), the unisensory visual (V) and the multisensory (AV) condition, in which A and V stimuli were presented simultaneously. Following previous procedures [10,12,14] a fourth condition was created summing up the ERP from the two unisensory conditions (A + V condition).

Since multisensory integration also involves sensory cortices and ERPs associated with sensory preparation seem to originate in the same areas affected by post-stimulus multisensory interaction, we hypothesize that the preparatory ERPs may also be affected in the same way as post-stimulus ERPs. Specifically, we predict larger vN and aP amplitudes in the multisensory condition (AV) than the sum of the unimodal condition (A + V), so that AV > A + V. If this hypothesis was confirmed, the amplitude difference (i.e., AV vs. A + V) would suggest a cross-modal interaction of sensory anticipation for upcoming events. If the anticipatory multisensory integration was confirmed, we will also test to what extent this anticipatory activity affects the following early post-stimulus AV integration.

Results from the present study would pave the way for new perspectives on multisensory integration in the normal brain and for possible clinical implications in neurological patients.

## 2. Materials and Methods

### 2.1. Participants

The *a priori* power analysis, performed using the G*Power 3.1.9.2 [29], showed that a minimum of 22 participants was required to reach the effect size (d) of 1, a power (1-β error probability) of 0.95, and a 0.01 alpha probability for the two-tailed dependent sample tests.

Twenty-four adults volunteered for this study (14 females, mean age 26.6 years SD = 8.8). The inclusion criterion was the absence of any reported neurological or psychological disorders. All participants had normal or corrected-to-normal vision and hearing and were naive about the aim of the study. On average, they showed preference for the right hand, which was evaluated by the Italian version of the Edinburgh Handedness Inventory Questionnaire [30]. Written informed consent was obtained from all participants according to the declaration of Helsinki; the project was approved by the Santa Lucia Foundation Ethical Committee.

### 2.2. Procedure

Participants were seated in front of a screen placed 114 cm from their eyes with their arms positioned palm down on the armrests. During the whole run, a fixation point was displayed in the centre of the computer screen and consisted of a yellow circle (diameter 0.15 × 0.15° of visual angle) on a black background. Participants were instructed to remain relaxed and inactive, to not cross their legs and arms during stimulus delivering, and to maintain their gaze on the fixation point during the run. In every run, 80 stimuli were randomly presented with a variable interstimulus interval (ISI) of 1–2 s, for a total duration of approximately two minutes. The used ISI has been proven to be variable enough to avoid activity overlap between the adjacent trial [31]; however, a control experiment (Experiment 1 in the Supplementary Material and Figure S1), which only used trials with ISIs longer or equal to 1.5 s, confirmed that 1–2 s ISI is not an issue. After each run, the participant could have a short break if needed. Participants were instructed to passively look at and/or hear the presented stimuli without performing any cognitive or motor task. Three conditions were randomly presented: auditory (A), visual (V), and audiovisual (AV). For each condition, four runs were performed, presenting a total of 320 stimuli in about 10 min. The conditions were counterbalanced across participants.

In the A condition, binaural sounds with a duration of 250 ms were presented through two loudspeakers placed symmetrically on each side of the computer screen. Sound volume was regulated to be perceived as pleasantly as possible on an individual basis (about 65–70 dB). The sounds consisted of four complex tones with the following features: 10 ms rise and fall, 16 harmonic components, 4410 Hz sample rate, 16-bit sound depth, stereo master, 60 dB SPL, and WAV audio file format. The software *Praat* (www.fon.hum.uva.nl/praat, accessed on 11 February 2019) was used for sound synthesis. The four sounds were composed of the following fundamental frequencies: 740 Hz (i.e., F#5), 1046 Hz (i.e., C6), 2093 Hz (i.e., C7), and 2960 Hz (i.e., F#7).

In the V condition, stimuli consisted of one of four squared (4 × 4° of visual angle) geometrical configurations composed of vertical and horizonal lines, which were presented for 250 ms each.

The described stimuli for each condition were chosen for their neutral and abstract shape in order to avoid any emotional or attentional bias in the sensory processing.

**Figure 1:**
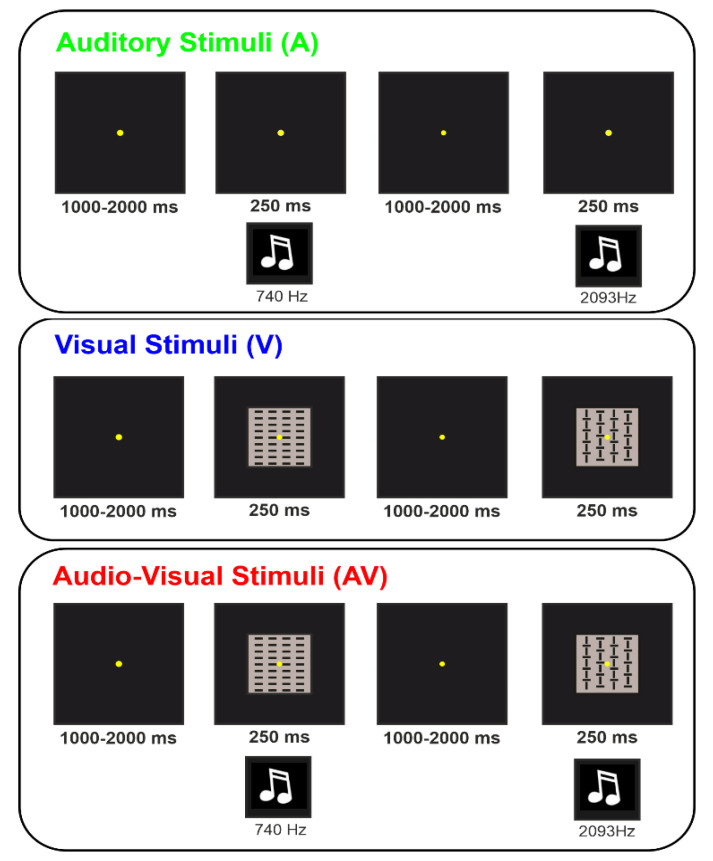
Schematic representation of the stimuli adopted in the three conditions for the Auditory (A), Visual (V) and Audio-Visual (AV) modalities. In the AV condition, auditory and visual stimuli were presented simultaneously. Two examples of the combination between stimuli in the AV condition are displayed in the Figure.

In the AV condition, A and V stimuli were simultaneously administered. For details on A and V stimuli, please refer to the unisensory modalities. In order to ensure a proper comparability between the present and the previous studies using similar auditory and visual unimodal stimulations [26,27], we used the same visual and auditory stimulus coupling. The four stimulus categories in each condition were presented randomly with equal probability (*p* = 0.25). Figure 1 shows a representation of the three conditions.

### 2.3. EEG Recording and Analysis

All participants were individually tested in a dim, sound-attenuated room using a 96-channel EEG system (Brainamp™ amplifiers) with 64 active scalp electrodes (Acticap™) and Recorder 1.2 and Analyzer 2.2 software, all by BrainProducts GmbH (Munich, Germany). The electrodes were mounted according to the 10-10 International System, initially referenced to the left mastoid (M1), and then off-line re-referenced to the M1-M2 average. Horizontal and vertical electrooculograms (HEOG and VEOG) were monitored by bipolar recordings, with electrodes positioned at the left and right external canthi (HEOG) and below and above the left eye (VEOG). EEG was amplified, digitized (250 Hz), filtered (0.01–60 Hz bandpass with a 50 Hz notch filter) and stored for further analysis. EEG was further filtered off-line (0.1–30 Hz bandpass) and processed to reduce ocular artifacts using independent component analysis (ICA) available in the Analyzer software. Semi-automatic artifact rejection was performed prior to signal averaging to discard epochs contaminated by other signals exceeding the amplitude threshold of ±70 μV. The filtered signal was then segmented in 1300 ms epochs, starting from −1100 ms pre to 200 ms post stimulus onset. Pre- and post-stimulus ERP components were measured with respect to a −1100/−900 ms and −200/0 ms baseline, respectively.

In addition to the mentioned three conditions, a fourth condition was made summing up the A and the V conditions at the individual level, which was labelled A + V. Finally, four grand-average ERPs (i.e., group averages) were obtained for each experimental condition.

To select the intervals and electrodes to be considered in statistical analysis, the “collapsed localizer” method was used [32], in which a localizer ERP is obtained by collapsing (averaging) all experimental conditions. To identify the interval of analysis, the global field power (GFP) was calculated. The GFP describes the ERP spatial variability at each time point considering all scalp electrodes simultaneously, resulting in a reference-independent descriptor of the potential field. The pre-stimulus interval in which the GPF was larger than 80% of its maximum value was used for further analysis. The same calculation was also made for the early post-stimulus interval (0–200 ms) to identify the auditory N1 and the visual P1 components. The GFP approach selected one pre-stimulus interval from −500 ms to 0 ms and one post-stimulus interval from 100 to 132 ms, in which the mean amplitude was calculated for statistical purposes. The electrodes with an amplitude larger than 80% of the maximum value in the intervals selected by the collapsed localizer were jointed in spatial pools and considered for statistical analysis. Two foci of activity were clearly present: the medial frontal activity of the aP and the N1, and the bilateral parieto-occipital activity of the vN and the P1 components. The aP and the N1 were then represented by a pool containing AFz, F1, Fz, F2 and FCz electrodes (frontal pool). The vN and the P1 were represented by a pool containing PO9, PO7, O1, O2, PO8 and PO10 electrodes (parieto-occipital pool).

The onset latency of the vN and the aP were also calculated in the A, V and AV conditions as the first time point exceeding more than twice the absolute value of the averaged 200 ms baseline at vN and aP peak electrodes (O2 and Fz, respectively).

Statistical analysis was performed using two tailed t-tests for dependent samples, comparing the AV and A + V conditions for onset latency, the mean amplitude of the aP and vN components, and the mean amplitude of the N1 and the P1 components. We also performed topographical t-tests, comparing each single electrode to reach a broader understanding of the two conditions’ differences. For t-tests on the electrode pools, the alpha threshold was set to 0.05. For topographical t-tests, the alpha level was set to 0.01 to compensate for the multiple comparisons. Cohen’s d (d) was also reported as a measure of effect size. At first, Levene’s and Wilk-Shapiro’s tests for equality of variance and normal distribution, respectively, were performed, showing no violation of the sample homoscedasticity and distribution. All statistical analyses were performed using the Statistica 12.0 software (StatSoftinc., Tulsa, OK, USA).

## 3. Results

**Figure 2:**
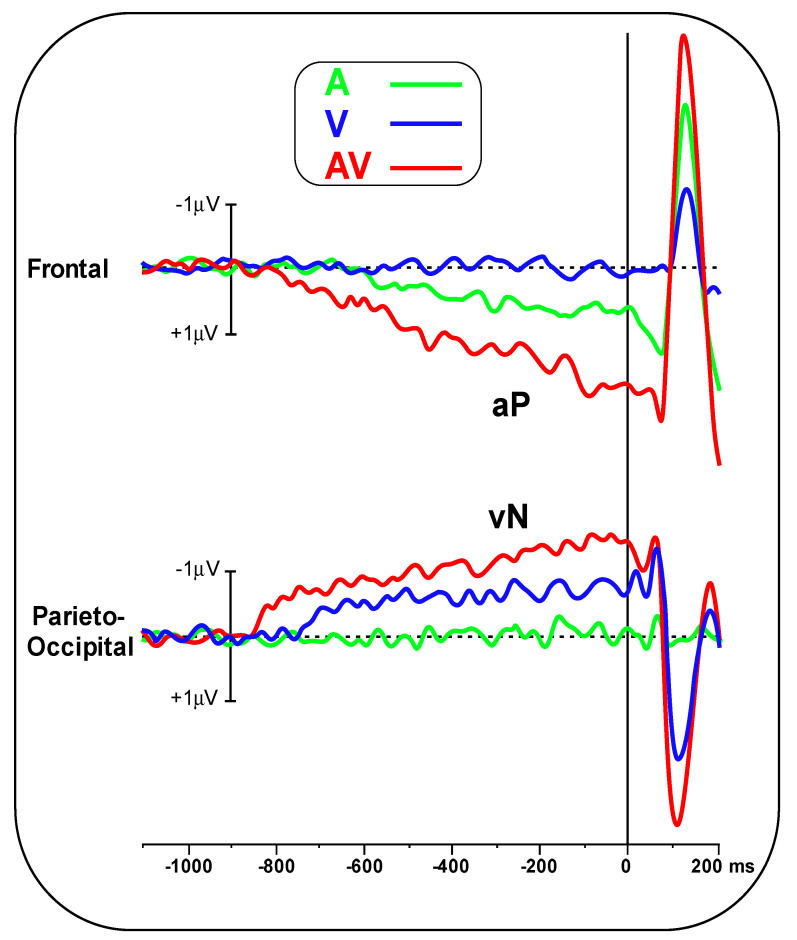
Pre-stimulus ERP in the A, V and AV conditions at the frontal pool, showing the aP component, and the parieto-occipital pool, showing the vN.

**Figure 3:**
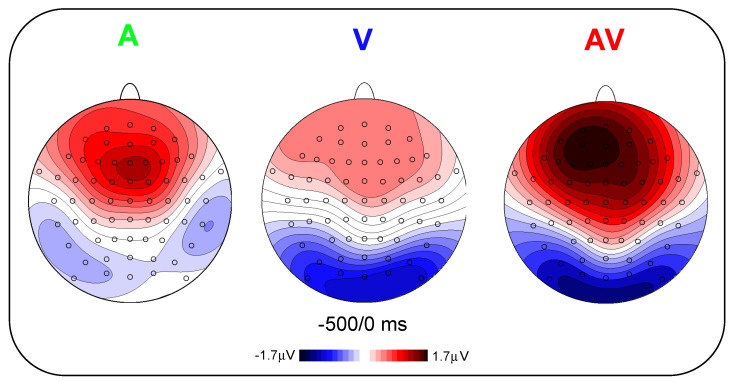
Topographic distribution of the average activity of pre-stimulus ERPs from −500 to 0 ms in the three experimental conditions.

Figure 2 shows the pre-stimulus waveforms for the A, V and AV conditions at the frontal and parieto-occipital pools. Figure 3 shows the scalp topography (top-flat view) in the three conditions in the −500/0 ms interval. In the A and AV conditions, a positive slow-rising voltage shift emerged over the medial frontal sites 700–800 ms before stimulus onset and peaked right after it. This positivity was accompanied by two small bilateral temporal foci, typical of bilateral auditory cortices activation. This component has been labelled as auditory positivity, or aP [26], and it was absent in the V condition. In the V and AV conditions, a slow negative wave initiated at around −800 ms and was prominent over bilateral parieto-occipital areas with the typical distribution of extrastriate visual areas. This component has been labelled as visual negativity, or vN [26], and it was absent in the A condition.

The onset latency of the aP in the A condition was −670 ± 102 ms, while in the AV condition, it was −790 ± 137 ms. Onset latency of the vN in the V condition was −740 ± 108 ms, while in the AV condition, it was −850 ± 134 ms. Statistical analysis of aP onset latency showed an earlier amplitude for the AV condition (t_(23)_ = 3.44, *p* = 0.002, d = 0.99). The same result was found for the vN (t_(23)_ = 3.132, *p* = 0.0047, d = 0.90).

**Figure 4:**
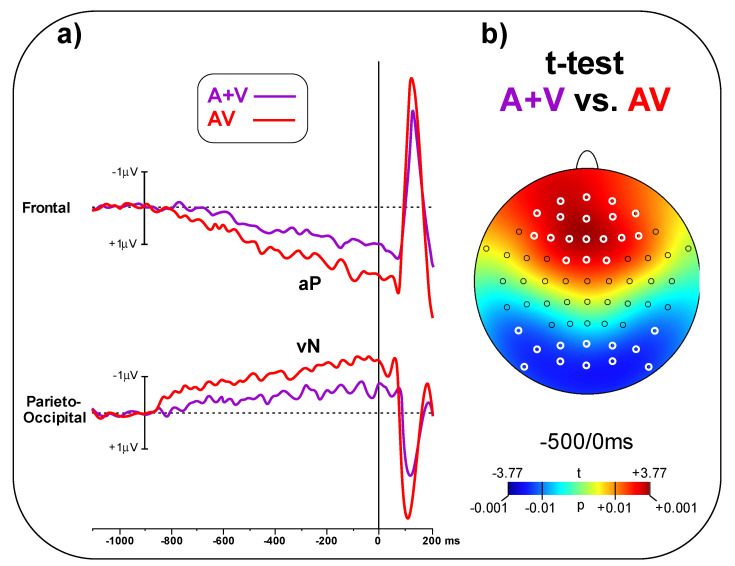
(**a**) Simultaneous (AV) and summed (A + V) pre-stimulus ERPs can be observed in the two electrode pools. (**b**) Statistical map depicting the t-test topographical distribution. Electrode scoring (t < 0.01) is highlighted in white.

In Figure 4a, the A + V and AV conditions are compared, showing larger amplitude for the AV than the A + V condition in the pre-stimulus phase. The t-test on the frontal pool showed significant differences (t_(23)_ = 3.48, *p* = 0.002, d = 1.00) between conditions (AV = 1.65 ± 0.98 μV, A + V = 0.82 ± 0.64 μV). Similarly, the t-test on the parieto-occipital pool indicated a significant difference (t_(23)_ = 2.94, p = 0.007, d = 0.85) between conditions (AV = 1.23 ± 0.68 μV, A + V = 0.72 ± 0.51 μV). Figure 4b shows the topographical t-test on all the scalp electrodes, indicating that significant differences between AV and A + V go well beyond the selected electrodes pools; the aP effect involved 18 central-frontal electrodes, while the vN effect involved 12 parieto-occipital electrodes. Significant electrodes are highlight in white in Figure 4b.

**Figure 5:**
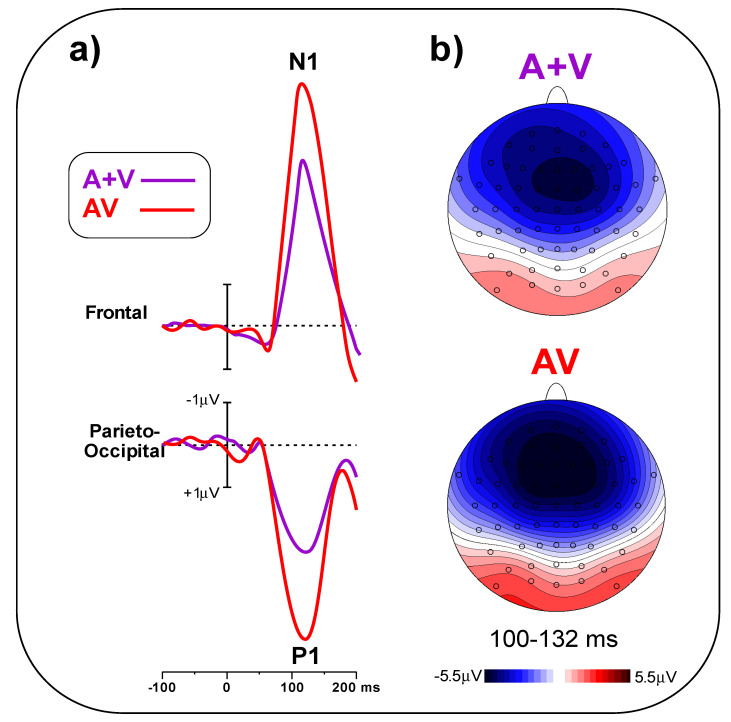
(**a**) AV and A + V post-stimulus ERPs in the frontal and parieto-occipital pools. (**b**) Topographical distribution of the P1 and the N1 components in the 100–132 ms interval.

In Figure 5a, early post-stimulus ERPs in the A + V and AV conditions are compared, indicating a larger amplitude for the AV than the A + V condition. The t-test on the frontal pool showed significant differences (t_(23)_ = 5.25, *p* < 0.001, d = 1.52) between conditions (AV = 5.49 ± 1.28 μV, A + V = 3.78 ± 0.95 μV). Similarly, the t-test on the parieto-occipital pool indicated a significant difference (t_(23)_ = 6.09, *p* < 0.001, d = 1.97) between conditions (AV = 4.47 ± 1.26 μV, A + V = 2.58 ± 0.85 μV). Figure 5b shows the scalp topography of the N1 and P1 components in the 100–132 ms interval.

Comparing Figure 4a and Figure 5a, it is worth pointing out that part of the post-stimulus AV effect is due to the pre-stimulus effect. Indeed, the post-stimulus ERPs’ baseline is typically calculated in the last 100–200 ms before stimulus onset [33], which, compared with the −1100/−900 ms baseline used for the pre-stimulus analysis, “resets” the aP and vN amplitudes (and the pre-stimulus AV effect) and enhances the N1 and P1 amplitude and the relative AV effect. Therefore, in order to reliably assess to what extent the pre-stimulus AV effect actually explains the post-stimulus AV effect, we subtracted from the N1 and P1 effects (regularly calculated with the −200/0 ms baseline) with those obtained using the −1100/−900 ms baseline. From these analyses, we observed that the pre-stimulus effect explains about 50% of the post-stimulus AV effect. In detail, the −1.71 μV of the N1 effect is partially due to the 0.82 μV contribution from the aP effect, while the 1.89 μV of the P1 effect is partially due to the 0.94 μV contribution of the vN effect.

## 4. Discussion

In the present study, pre-stimulus ERPs were recorded in two unimodal passive sensory tasks (auditory and visual) and in a bimodal audio-visual task. ERPs obtained from the two unimodal tasks were then summed up and compared with the AV condition in order to detect possible sensory-specific anticipatory activities due to multisensory cortical integration in modality-specific sensory areas. Specifically, our aim was to test whether there was a difference in both the onset latency and the amplitude between the summed unimodal and the multisensory condition, considering the recently discovered pre-stimulus ERP components: the aP and the vN [25,26]. The results showed that our hypothesis was confirmed: in fact, both components have a significantly larger amplitude and earlier onset when stimuli are presented simultaneously (bimodal) than when they are presented as a sum of unimodal conditions. Therefore, a larger and earlier sensory preparation would indicate the occurrence of cross-modality interactions when bimodal, rather than unimodal, stimuli are expected. These findings suggest that, given the inevitable multisensory nature of the real-life environment, our brain seems to prepare in advance for multiple sources of sensory information in order to facilitate perception.

Further, in addition to confirming multisensory integration affects early post-stimulus reactive processing in passive tasks, as indexed by the N1 and P1 components enhancement [8,11,12,13,14,15,16], we showed for the first time that even perspectives of multisensory stimulation activate top-down processing in unimodal sensory areas. Crucially, since the time interval typically used as a baseline correction for post-stimulus activities also corresponds with the aP and the vN peaks, we also demonstrated that part of the N1 and the P1 AV effect is due to these anticipatory activities, which, given the opposite polarity, boost their effects on the post-stimulus stage. Collectively, the present data not only add novel knowledge to the growing literature on the spatiotemporal properties of cortical multisensory processing in humans, but they also stress the crucial importance of pre-stimulus anticipatory brain activities, which seem to deeply impact post-stimulus processing.

In light of the present findings and of a previous study showing the correlation between sensory readiness components, such as the vN, and RTs (the more negative the vN amplitude, the lower the RT) [25], we might propose that the “redundant target effect”, the phenomenon in which subjects tend to respond faster and better to multimodal stimuli compared to unimodal ones [34,35], may also in part depend on these preparatory components. Indeed, it is plausible to assume that our brain prepares itself to deal with every possible upcoming event (hardly unimodal). In fact, it might be evolutionarily useful to have an anticipatory endogenous system specialized for cross-modal preparation. This system would allow us to have an improved perception and presumably quicker responses to multimodal stimuli. Future research is encouraged to test the presence of preparatory ERP in multisensory motor tasks to disentangle the role of these sensory readiness components (aP and vN) from anticipating sensory-motor tasks (as the Bereitschaftspotential—or BP—and the prefrontal negativity—or pN—associated with motor and cognitive preparation, respectively [36]). Further, our study presents appealing findings in the context of brain–computer interface (BCI) applications, in which these slow cortical potentials might represent reliable BCI input signals in neurological patients, such as those who are unable to speak or move [37]. Another possible application could concern autistic spectrum disorder (ASD), given that 70–80% of autistic individuals show sensory abnormalities [38,39], and, according to the “central coherence theory” [40] social impairments could also depend on a lack of proper multisensory integration mechanisms. According to this theory, people with autistic symptoms tend to focus more on individual pieces of information (i.e., local details) at the expense of perceiving the wider picture [41]. Considering this, further research about anticipatory multisensory interaction could explain and give us a deeper knowledge of the neural bases of autistic spectrum disorders.

A study limitation can be represented by the possible interaction between anticipation intended as expectation and attention, which are intrinsic yet distinct components of the perceptual network [42,43,44]. Even in passive tasks, such as the present one, selective attention is directed to the stimuli and may interact with sensory readiness [45]. Future studies should try to disentangle expectation and attention using either unpredictable stimulus configurations or sensory motor tasks modulating attention.

Another limitation present in this study is the absence of an intermodal condition, which would alternate A, V or AV stimuli during the same task block, in order to confirm that the described result is linked to sensorial preparation and not to a habituation process. Furthermore, the use of fixed ipsimodal blocks avoids the “the modality shift effect” bias in multisensory integration studies [46]. To avoid this limitation, we made a control experiment (see Supplementary Material, control experiment 2 and Figure S2) to prove that the present findings could be attributed to a block factor that develops over time with repeated stimuli of the same A or V stimulus type and therefore to confirm that the anticipatory multisensory integration is a top-down process. Results of the control experiment showed no difference in the pre-stimulus component between the present blocked and the intermixed condition, confirming that the found anticipatory multisensory effect can be safety credited to top-down processes.

## 5. Conclusions

In conclusion, in this ERP study, by means of passive sensory stimulation paradigms, we confirmed the presence of anticipatory multisensorial integration in unimodal brain areas, which seem to boost early stimulus processing and audio-visual integration.

## Data Availability

Data are available from the corresponding author upon request.

## Supplementary Materials

The following are available online at https://www.mdpi.com/article/10.3390/brainsci11070843/s1, Figure S1: Pre-stimulus ERP as in Figure 2, but obtained including in the average only trial with ISI larger Than 1.5 s; Figure S2: Pre-stimulus ERP in the blocked A and V conditions and in the intermixed A/V condition at the frontal pool identifying the aP component and at the parieto-occipital pool showing the vN.
