# Supplementary material for: Neural Basis of Anticipatory Multisensory Integration
Source: Brain Sci. 2021 Jun 25;11(7):843. doi: 10.3390/brainsci11070843 (PMC8301880; doi:10.3390/brainsci11070843)
Supplement: Supplementary file 1 [file brainsci-11-00843-s001.zip › brainsci-1214979-SI.pdf]

Supplementary material

Neural basis of anticipatory multisensory integration

Linda Fiorini, Marika Berchicci, Elena Mussini, Valentina Bianco, Stefania Lucia and Francesco Di Russo

Control experiments

Control experiment 1

In order to control that the findings in the main experiment could be attributed to a block factor that develops over time with repeated stimuli of the same Audio (A) or Visual (V) stimulus type, and therefore to confirm that the anticipatory multisensory integration is a top-down process, the following control experiment was done randomly intermixing A and V stimuli (A/V condition). If the result of this experiment will still show the presence of preparatory ERP components aP and vN, we can confirm their top-down nature and therefore the effect of multisensory integration on preparatory brain activity.

Control experiment 2

In order to control that the findings in the main experiment could not be attributed to overlap from adjacent trials because the variable interstimulus interval (ISI) of 1-2 s may not have been long enough, we removed trials with ISIs shorter than 1.5 s and rerun the analysis with about half of the trials. If the result of this experiment will confirm the main experiment, we can be assured that the ISI is not an issue.

Methods

Control experiment 1

Participants, stimuli, procedure and data analysis were the same as in the main experiment, but instead of continuous A or V stimuli within the same block, an intermodal stimulation was displayed. In this condition A and V stimuli were randomly intermixed in each block (80 per run), with a total of 320 stimuli for each category.

Statistical analysis was performed using a t-test comparing the amplitude of the aP and the vN components in the relative blocked A or V condition of the main experiment with those obtained in

the intermixed AV stimulation of the intermodal condition. In this condition, the aP was measured at the frontal pool and the vN at parieto-occipital pool.

Control experiment 2

Participants, stimuli, procedure and data analysis were the same as in the main experiment, but instead of averaging the individual ERP using all the available trials, we only used the trials with ISIs shorter than 1.5 s (160 out 320 trials).

Results

Control experiment 1

Supplementary Figure S1 shows the pre-stimulus waveforms for the three conditions (blocked A and V, and intermixed A or V stimuli) at frontal and parieto-occipital pools. Even though the intermixed condition showed lower amplitude than the blocked condition, the t-test on both the aP and the vN showed no significant differences ($t_{(23)}$=1.7, p=0.103 for the aP and $t_{(23)}$=1.9, p=0.070 for the vN).

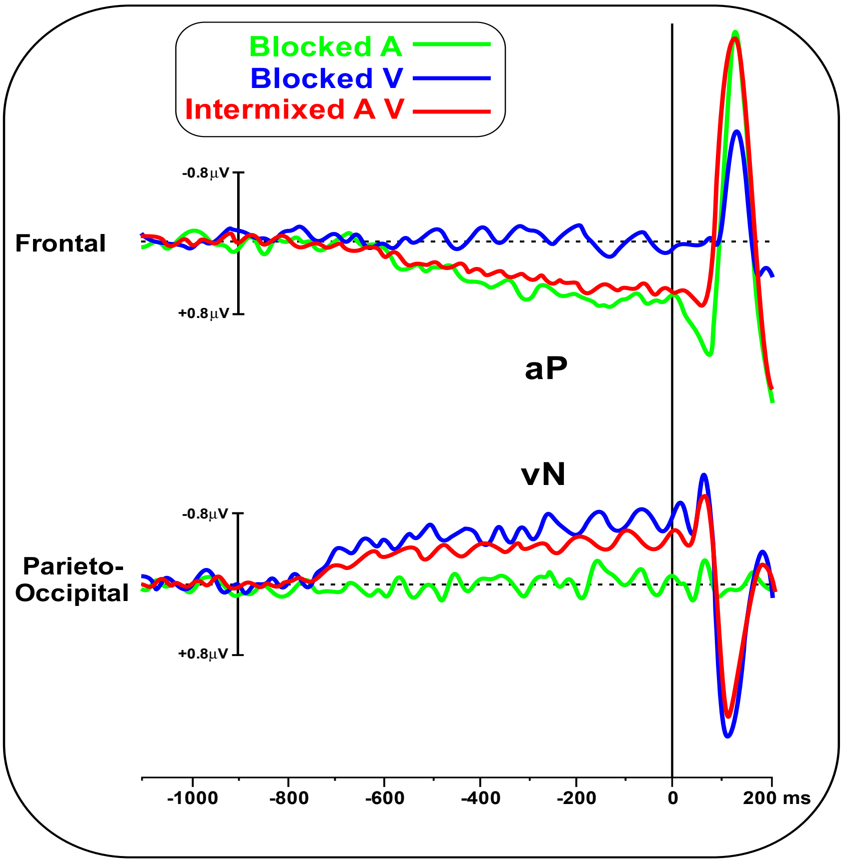

Supplementary Figure S1. Pre-stimulus ERP in the blocked A and V conditions and in the intermixed A/V condition at the frontal pool identifying the aP component and at the parieto-occipital pool showing the vN.

Control experiment 2

Supplementary Figure S2 shows the pre-stimulus waveforms for the three conditions (A, V and AV) at frontal and parieto-occipital pools as in the Figure 2 of the main experiment. Halving the trial clearly reduced the signal to noise ratio, but the pattern found in the main experiment remained unchanged. Statistical analysis confirmed the results showing on the frontal pool significant differences ($t_{(23)}$=3.12, p=0.005, d=0.94) between conditions. Similarly, the t-test on the parieto-occipital pool indicated a significant difference ($t_{(23)}$=2.94, p=0.011, d=0.81) between conditions.

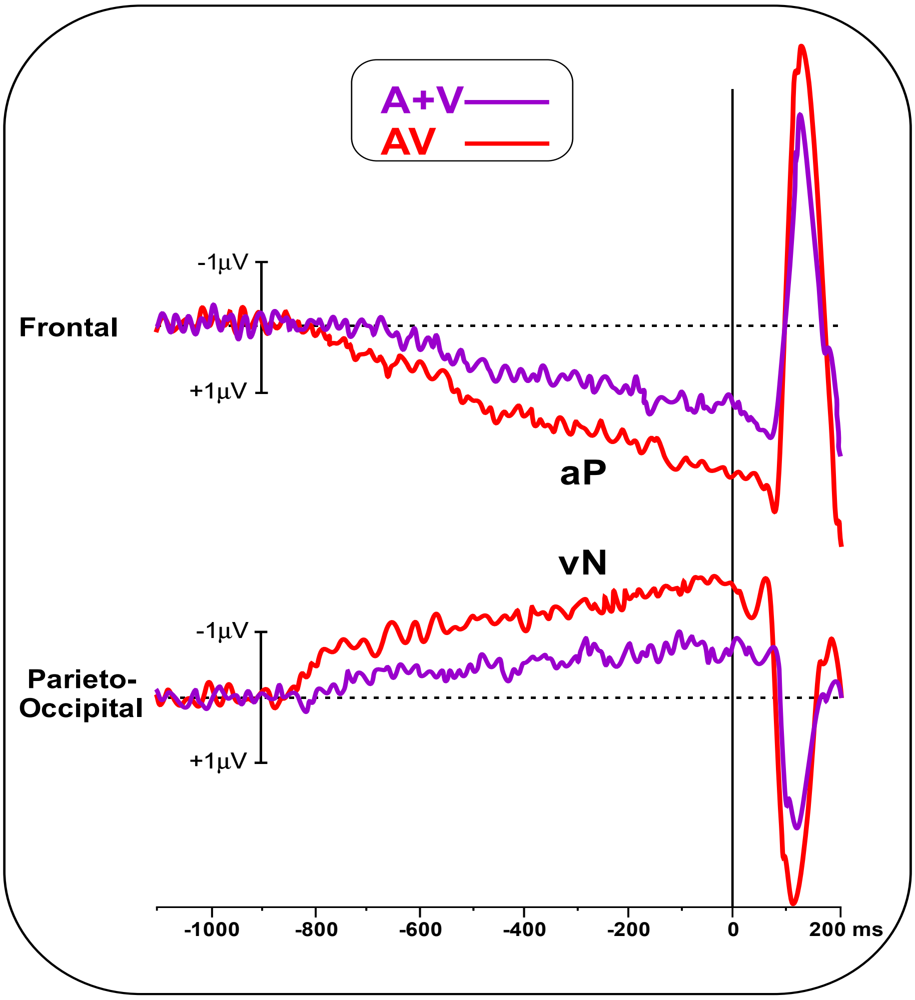

Supplementary Figure S2. Pre-stimulus ERP as in Figure 2, but obtained including in the average only trial with ISI larger Than 1.5 s.

Discussion

Results of the control experiment 1 showed that using random auditory or visual presentation (A/V condition), the aP and the vN components are simultaneously present as in the AV condition of the main experiment and they are comparable with those in the blocked conditions.

These results indicated that the findings of the main experiment cannot be attributed to a block factor that develops over time with repeated stimuli of the same A or V type. Therefore, the found anticipatory multisensory effect can be safety credited to top-down processes.

Results of the control experiment 2 showed that including in the ERP average only trials with ISIs shorter than 1.5 s the results remain unchanged, therefore, the used variable ISI (1-2 s) is not an issue.
